# Cold Plasma-Based Fabrication and Characterization of Active Films Containing Different Types of *Myristica fragrans* Essential Oil Emulsion

**DOI:** 10.3390/polym14081618

**Published:** 2022-04-16

**Authors:** Bara Yudhistira, Andi Syahrullah Sulaimana, Fuangfah Punthi, Chao-Kai Chang, Chun-Ta Lung, Shella Permatasari Santoso, Mohsen Gavahian, Chang-Wei Hsieh

**Affiliations:** 1 Department of Food Science and Biotechnology, National Chung Hsing University, Taichung City 40227, Taiwan; barayudhistira@staff.uns.ac.id (B.Y.); fuangfahp3@gmail.com (F.P.); kai70219@nchu.edu.tw (C.-K.C.); as920227@gmail.com (C.-T.L.); 2 Department of Food Science and Technology, Sebelas Maret University, Surakarta City 57126, Indonesia; 3 Department of Agro-Industrial Technology, Universitas Gadjah Mada, Yogyakarta 55281, Indonesia; andisyahrullahs@mail.ugm.ac.id; 4 Department of Chemical Engineering, Widya Mandala Surabaya Catholic University, Surabaya 60114, Indonesia; shella@ukwms.ac.id; 5 Department of Chemical Engineering, National Taiwan University of Science and Technology, Taipei 10607, Taiwan; 6 Department of Food Science, National Pingtung University of Science and Technology, Pingtung 91201, Taiwan; mohsengavahian@yahoo.com; 7 Department of Medical Research, China Medical University Hospital, Taichung City 40402, Taiwan

**Keywords:** active film, cold plasma, emulsion, essential oil, *Myristica fragrans*

## Abstract

*Myristica fragrans* essential oil (MFEO) is a potential active compound for application as an active packaging material. A new approach was developed using a cold plasma treatment to incorporate MFEO to improve the optical, physical, and bacterial inhibition properties of the film. The MFEO was added as coarse emulsion (CE), nanoemulsion (NE), and Pickering emulsion (PE) at different concentrations. The PE significantly affected (*p* < 0.05) the optical, physical, and chemical properties compared with CE and NE films. The addition of MFEO to low-density polyethylene (LDPE) film significantly reduced water vapor permeability (WVP) and oxygen permeability (OP) and showed marked activity against *E. coli* and *S. aureus* (*p* < 0.05). The release rate of PE films after 30 h was 70% lower than that of CE and NE films. Thus, it can be concluded that the fabrication of active packaging containing MFEO is a potential food packaging material.

## 1. Introduction

Active film is a new form of food packaging technology that includes functional additions to the packaging, such as antioxidants or antibacterial functions [1]; these are distinguished by the slow release of biologically active molecules into the food matrix over a long storage time [2]. Essential oils (EOs) are defined as volatile compounds, with preservation effects and several health benefits that can be isolated from plant materials through various techniques [3]; these are used in active packaging and have the status of “generally recognized as safe” [4]. *Myristica fragrans* (nutmeg) has a long history of use in traditional medicine as an antibiotic, antioxidant, and antithrombotic agent [5]. The active compounds in *M. fragrans* essential oil (MFEO) are sabinene, α-pinene, β-pinene, limonene [6], myristicin, and safrole and it contains high phenolic content [7]. Moreover, it has bacteriostatic properties [8]. Previous research by Balakrishnan et al. [9] showed that eugenol, isoelemicin, isoeugenol, methoxy eugenol, myristic acid, and myristicin from MFEO can be used for the fabrication of silver nanoparticles against MDR (multidrug-resistant) *Salmonella enterica*. MFEO has bacteriostatic properties as evidenced by very low concentrations that can inhibit bacteria and yeasts including *Arizona*, *Salmonella*, *Morganella*, *Entrobacter*, *Escherichia coli*, *Klebsia pesudomon*, *Pseudomonas aeruginosa*, *Staphylococcus aureus*, *Candida albicans*, *Cryptoccus neoformans*, *Aspergillus flavous*, *Tericophyton verruco*, and *Epidermophyton floccodum*. The highest inhibition effectiveness was on *Escherichia coli*. Based on a previous study, storage of Bovine loin (*Longissimus dorsi*) coated with an active film with polyvinyl alcohol, gelatin, and MFEO was shown to suppress the increase in total volatile nitrogen base and peroxide value and maintain color parameters, which in turn can extend the shelf life of meat [10]. It can be used as a potential candidate for inclusion in packaging.

According to Liu et al. [11], EOs have volatile compounds that readily evaporate during film formation and storage. The major challenges in incorporating EO into films are the development of poor miscibility and transparency, phase separation in the film production process, and the sensitivity of bioactive compounds to environmental factors [4]. Nanoencapsulation can cover the odor and taste of EOs and can mitigate the effect on food sensory properties and provide an effective distribution of the EO release properties [12]. Biopolymer-stabilized emulsions, referred to as Pickering emulsions (PEs), are interesting; the advantages of PEs include the increased stability to coalescence, increased load capacity, enhanced protection of the encapsulated component, and decreased release rate [2]. In a previous study, it was proven that the addition of PEs to a chitosan film increased the oxygen barrier property [13]. PEs have many advantages and are potential materials for use in packaging development to improve the function of packaging, particularly the protective effects and the release of active compounds.

EOs have drawbacks, including high volatility and sensitivity to oxidation, light, and thermal decomposition [14]. For this reason, efforts are needed to improve the application of EOs; one method uses different wall materials, including biopolymers. For the core material of PEs, protein complexation with polysaccharides can be used. Protein matrix filler agents can use carbohydrates that can protect active compounds [15]. In a previous study, the encapsulation of marjoram EO using inulin (a polysaccharide) with whey protein isolate in pectin film showed good mechanical and water barrier properties due to their highly dense and less permeable structure [16].

Cold plasma is an emerging technology that has been used for several purposes, including microbial inactivation and removal of hazardous chemicals [17]. It is an efficient, cost-effective, and ecologically acceptable means of substituting pollutants and dangerous coating techniques [18]. However, the application of this technology to develop essential oil-based packaging is a newly developed topic. The studies on active packaging prepared by cold plasma treatment use free EO or coarse emulsion (CE). Those studies are related to EOs in film, such as oregano EO [11], lemongrass EO [19], and marjoram EOs [16]. In addition, the use of active components from other natural sources has been investigated, including the use of cellulose nanofibers and filmogenic soy protein, which can improve the mechanical properties of the film [20] and the physicochemical properties of soy protein isolate-oil emulsion films are affected by oil droplets and the heating temperature of soy protein isolate [21], edible coating of whey protein isolate nanofibers and carvacrol showed antibacterial activity that can maintain the quality of salted duck egg yolk [22]. Furthermore, other active components that were investigated are exopolysaccharides from *Lactobacillus plantarum*, which show inhibitory properties of α-glucosidase and α-amylase and have antioxidant activity [23]. According to the above, the application of the PE system to packaging prepared by cold plasma treatment is still rare.

The addition of EO to the film is expected to increase the biological activity which necessitates research regarding the effect of adding external materials to improve film properties [24]. In this sense, the aim of this work was to use cold plasma to develop an active packaging containing MFEO and to characterize the properties of CEs, NEs, and PEs of MFEO-loaded LDPE films and to evaluate the effect of MFEO addition on the optical properties, physical properties, microbial inhibition characteristics, and release properties of LDPE films.

## 2. Materials and Methods

### 2.1. Materials

*Myristica fragrans* essential oil (MFEO) with a 95% purity was purchased from Pulau Pinang, Malaysia. The *Staphylococcus aureus* (*S. aureus*) strains 328 (BCRC 15211) and *Escherichia coli* (*E. coli* O1:K1:H7) strains NCTC 9001 (BCRC 10675) were purchased from Bioresource Collection and Research Centre (BCRC) of the Food Industry Research and Development Institute in Hsinchu, Taiwan. Chemical reagents were obtained from Sigma-Aldrich (St. Louis, MO, USA).

### 2.2. Preparation of Emulsion

CEs, NEs, and PEs were used in this study with MFEO concentrations of 1%, 3%, and 6%. The CEs and NEs were prepared using the method of Noori et al. [25]. The CE was prepared by the gradual addition of MFEO (1% wt) and Tween 80 (30% of MFEO) into distilled water with stirring at 314.16 rad/s. Nanoemulsification used a sonicator (Sonopuls HD 4200, Bandelin, Berlin, Germany) to produce NEs operating at 20 kHz and 200 W.

The method of Hosseinnia et al. [15] was used to prepare WPI/inulin-stabilized PE. A ratio of 1:1 for WPI (2% wt) and inulin (2% wt) in distilled water was used as the biopolymer suspension. Then, 1 g of MFEO was added slowly to the biopolymer dispersion with stirring at 523.60 rad/s to obtain a pre-emulsion with a core-coating ratio of 1:4. Ultrasonication was conducted for 20 min at room temperature to the prepare PE, which was then dried using a freeze dryer (FD50-6S-S, Kingmech, New Taipei, Taiwan) at 0.007 atm and −40 °C for 48 h. The PE was stored in an airtight container that was dark in color and stored in a refrigerator.

Dynamic light scattering was used to assess the diameter and polydispersity index (PdI) of droplets (Zetasizer Nano ZS-90, Malvern Instruments, Worcestershire, UK) [19].

### 2.3. Preparation of LDPE-Treated Film

LDPE-treated film was prepared using the method of Wong et al. [26]. The films were first cut into 7 × 9 cm and cleaned using 75% alcohol. A vacuum plasma reactor uses a cold radiofrequency plasma (13.56 MHz) (Model 1000W, Junsun Tech Co., New Taipei, Taiwan) and a pressure of 0.0643 Torr. The film was treated for 60 s at a power of 30 W. After the nanocarriers were mixed using a magnetic stirrer (15 min) [16], the CE, NE, and PE solutions (1 mL) were then evenly coated on plasma-treated LDPE films. The solution was spread evenly over the entire surface of the LDPE film using a glass tu stick triangle dish and dried for approximately 24 h. The control was an LDPE film without plasma treatment and without coating of MFEO [27].

### 2.4. Optical Properties

The method of Mendes et al. [19] was used to determine the optical characteristics (*L*, *a*, *b*, and yellowness index (*YI*)) of the film using a color difference meter (ZE6000, Nippon Denshoku Co., Tokyo, Japan). The following formulas were used:

(1) $$\Delta E=\sqrt{{\Delta L}^{2}+{\Delta a}^{2}+{\Delta b}^{2}}$$

(2) YI=(142.86×b)/L

Film opacity was determined using a UV-visible spectrophotometer (CT- 8600, Chrom Tech, Taipei, Taiwan) at an absorbance of 600 nm [28].

The water contact angle (WCA) was measured in accordance with the method of Grzegorzewski et al. [29] and was determined using a water contact angle instrument (Si-plasma CAM-120, Creating Nano Tech., Tainan, Taiwan). Drops of liquid distilled water were dispersed on the adaxial surface of each film using a microliter pipette. WCA analysis was performed in five different measurement positions for each film.

### 2.5. Physical and Mechanical Properties

Film thickness was measured to the closest 0.001 mm using a hand-held digital micrometer (Mitutoyo 293-185-30 Quantumike, Digimatic, Elgoibar, Spain).

The texture profile analyzer (TA-XT2i, Stable Micro Systems Ltd., Surrey, UK) detected the film’s tensile strength (TS). A load cell of 500 N and a gauge length of 100 mm were used in line with the ASTM D882-91 standard test procedure [30]. Shearing strength measurements were performed on a texture profile analyzer (TA-XT2i, Stable Micro Systems Ltd., Surrey, UK) with a load cell of 40 N and a gauge length of 80 mm. Tensile and shear strength were determined using three replications of the sample and analysis.

(3) Tensile strength=F/(W×D)

where F is the force required to break the film (N), W denotes the film width (mm), and D denotes the film thickness (mm).

The film water vapor permeability (WVP) was determined using the ASTM E96 standard. The film was placed in an aluminum cell containing silica gel and placed in a desiccator containing distilled water (100% RH, 30 °C). The aluminum cells were weighed from day 0 to day 10 to ensure steady-state permeation [31]. The following formula was used:

(4) WVP=Δg/Δt (x/A×ΔP)

where Δg/Δt is the rate of weight change (g/h), x is the film thickness (mm), A is the permeation area (0.0032 m^2^), and ΔP is the partial pressure difference of water vapor saturation across the film (4244.9 Pa at 30 °C).

The deoxidizer absorption method was used to measure the film oxygen permeability (OP). The bottle was filled with a deoxidizing agent (3 g) and the film was placed on the bottle and closed. The bottles were weighed before being put in a desiccator at a temperature of 23 °C with 75% RH for 48 h [32]. The following formula was used:

(5) OP=(Δm×d)/(A×t×P)

where Δm is the weight variation in the test bottle (kg), d is the thickness of the film (m), A is the effective area of the film (m^2^), t is the time interval (s), and P is the partial pressure difference of oxygen on both sides of the film (Pa).

An attenuated total reflection Fourier transform infrared spectroscopy (Thermo Nicolet 6700 ATR-FTIR, Thermo Fisher Scientific, Taichung, Taiwan) was used to determine the chemical composition of the film surface [30]. A scanning electron microscopy (SEM) (JEOL JSM-7800F Prime Schottky) was performed for the evaluation of film morphology [26].

### 2.6. Antioxidant Properties

The DPPH (2,2-diphenyl-1-picrylhydrazyl) method was used to measure the antioxidant activity of the films [33] with slight modifications [26]. A total of 35 mg of film was dissolved in 3 mL of distilled water, and then 1 mM of DPPH methanol solution was added and reacted for 30 min in the dark. The following formula was used:

(6) $$\mathrm{DPPH}\;\mathrm{scavenging}\;\left(\%\right)=\left({\mathrm{abs}}_{\mathrm{DPPH}}-{\mathrm{abs}}_{\mathrm{extract}}\right)/{\mathrm{abs}}_{\mathrm{DPPH}})$$

The total phenolic content (TPC) was measured by weighing 100 mg of film and soaking it in 10 mL of distilled water prior to incubation for 3 h [34]. Then, 2 mL of the supernatant was analyzed using the Folin–Ciocalteu method. The solution was homogenized and determined using a UV-visible spectrophotometer (CT- 8600, Chrom Tech, Taipei, Taiwan) at 725 nm. The TPC was expressed in mg gallic acid equivalent per 100 g sample [35].

### 2.7. Antibacterial Assay

*Staphylococcus aureus* (*S. aureus*) and *Escherichia coli* (*E. coli*) were used to examine the inhibitory effect of the film on bacterial growth. The cells were cultured by inoculating 100 μL from 10 mL of nutrient broth pre-cultures and incubated for 16–18 h. Each bacterial culture was adjusted to a cell concentration of 10^5^ CFU/mL using Mueller–Hinton broth. Each film was dipped into 10 mL of cell suspension in the microbe and shaken at 150 rpm. Finally, 100 μL of the suspension was placed on plate count agar and stored at 37 °C for 24 h [36].

### 2.8. Release Properties

The MFEO release was measured from a film (2 × 2 cm^2^) inserted into a vial containing 95% ethanol (10 mL) and the vials were placed in the dark at room temperature for 34 h. Then, 1 mL of solution was examined using a UV-visible spectrophotometer (CT-8600, Chrom Tech, Taipei, Taiwan) at 329 nm (with slightly modification) [1,16]. The absorbance of the sample was compared with the total absorbance of the film until a constant absorbance was reached, indicating that all active compounds were released to the simulant. The following formula was used:

(7) $$\mathrm{Release}\;\mathrm{rate}\;\left(\%\right)=\left({\mathrm{abs}}_{\mathrm{total}}-{\mathrm{abs}}_{\mathrm{sample}}\right)/{\mathrm{abs}}_{\mathrm{sample}}\;$$

The Higuchi and Korsmeyer–Peppas equations were used to fit cumulative release data over time to determine the EO’s release kinetics. The following formula was used:

(8) $$\mathrm{Higuchi}\;:\;\frac{\mathrm{Mt}}{M\infty }=K_{1}t^{1/2}\;$$

(9) $$\mathrm{Korsmeyer}-\mathrm{Peppas}\;:\;\frac{\mathrm{Mt}}{M\infty }=K_{2}t^{n}\;$$

where Mt/M∞ is the percentage of EOs released at time t; k_1_ and k_2_ are constant characteristics of the bioactive-polymer system; n is the diffusion index, which denotes the parameters relating to the mechanism of release.

### 2.9. Statistical Analysis

The mean ± standard deviation is shown for all data. The statistical analysis was calculated using SPSS (version 20) software with one-way ANOVA and Duncan’s multiple-range test (significance *p* < 0.05). All parameters used three replicates for sample and analysis.

## 3. Results and Discussions

### 3.1. Emulsion Properties

The MFEO concentrations were maintained at constant levels (1%, 3%, and 6%); based on Shokri et al. [37], 3% of *Ferulago angulata* EO had good bacterial inhibition but poor antioxidant activity. Therefore, in this study, an attempt was made to increase the concentration of EO. As shown in Table 1, the CE droplet size was larger and significantly different from NE and PE (*p* < 0.05). It can be seen that the CE treatment has the same subset as the NE and PE treatments. The droplet size for all samples is in the range of 170.93–1731.23 nm, where NE and PE have droplet sizes below 250 nm. Emulsion stability in a film matrix is indicated by the small droplet size. Furthermore, it can reduce the rate of droplet aggregation, flocculation, and coalescence [11]. In addition, NE has the advantage of more stability and more transparency [16] based on the zeta potential parameters (Table 1) for all samples are in the range of 8.47–40.80 mV. The difference in the type of emulsion and the concentration of MFEO had a significant effect on the zeta potential (*p* < 0.05). In the CE treatment, there was a significant difference in the level of each different concentration (*p* < 0.05), while in the NE and PE treatments, the relative zeta potentials were not so different. In a previous study, increasing the concentration of *Grammosciadium ptrocarpum* Bioss. EO (GEO) decreased the zeta potential [1] and the NE has a lower zeta potential than the CE [25]. Zeta potential estimates the interaction and surface charge characteristics at the molecular level, where an emulsion with a potential zeta value of ≤−30 mV or ≥+30 mV provides emulsion stability [38]. In a previous study, ultrasonic treatment of 300 W for 20 min on microgel particles can provide the highest potential. This is because ultrasonic treatment causes a redistribution of charged chains on the particle surface so that the interfacial and internal structures change [39]. PE can be stabilized by whey protein isolate nanofibrils where oil droplets can be dissolved and wrapped by whey protein isolate nanofibrils to prevent oil droplets from coalescing. This is because fibrillation increases the zeta potential and flexibility of whey protein isolate nanofibrils, allowing them to self-assemble at the oil-water interface to form a layer with higher electrostatic repulsion and the stretched structure can have more intermolecular hydrogen bonds and Van der Waals force, enhancing the interface layer’s rigidity and preventing oil droplet coalescence, stabilizing the PE [40]. In this study, it was shown that the stable emulsion was only in CE 1% and CE 3% samples. This is possible because the power and time of ultrasonication in the production of each emulsion use the same power and time, so it is necessary to optimize the power and time of ultrasonication to obtain an emulsion with a zeta potential that leads to a stable emulsion.

As shown in Table 1, the PdI parameters in the CE treatment resulted in a significant difference compared with NE and PE (*p* < 0.05). In the CE treatment, the PdI did not differ significantly for different MFEO concentrations, while the PE treatment had the lowest PdI, especially at 3% and 6% MFEO concentrations. In a previous study, the PdI of ginger EO emulsion at NE (0.222) was lower than at CE (0.584). Ultrasonication produces a lower PdI in NE and a uniform particle size [25]. Emulsions that have a PdI lower than 0.3 indicate a stable emulsion and have uniformity of emulsion droplets [16].

### 3.2. Optical Properties

As shown in Table 2, the *L* value of the treated film was significantly lower than that of the control film (*p* < 0.05). In the color parameters, especially *L* and *a*, it can be seen that the film with PE 6% and NE 6% treatment had the highest value and was significantly different from the film with other treatments. In addition, the increase in MFEO concentration significantly increased the red–green (*a*) and blue–yellow (*b*) values (*p* < 0.05) (Table 2). An increase in EO causes the film to become more yellow. The ΔE in all the treated films was less than 2, indicating only a slight difference in film appearance [19]. PE films of marjoram essential oil (MEO) had higher ΔE than NE films [16], this demonstrated that emulsion type and concentration affect the color of films. The *YI* increased as the MFEO concentration increased and was higher than the control film (*p* < 0.05) (Table 2). This shows something similar to the color parameter at the *b* value. In this study, in PE films the increase in yellowness was due to the natural bright yellow color of the whey protein isolate. This is because the type and concentration of EOs, as well as the presence of additives, directly affect the color of the emulsified film [16].

In this study, the opacity of all film samples was significantly higher than the control (Table 2). The opacity parameter for films with 6% PE treatment has the highest value and is significantly different from other treatments. An increase in the concentration of MFEO caused an increase in the opacity. A high opacity indicates that the films have low transparency. The PE films had the largest opacity because there was a matrix formed from inulin and WPI, and the existence of an oil phase in the protein matrix increases light dispersion and the light-scattering impact of the oil [41].

From Table 2, it also can be seen that an increase in the MFEO concentration causes an increase in the WCA. As in the opacity parameter, the film with PE 6% treatment had the highest WCA value and was significantly different from other treatments. The incorporation of hydrophobic compounds and plasma treatment increases the surface hydrophobicity [26,27]. However, according to Liu et al. [11], WCA is not only related to surface hydrophobicity but also influenced by porosity and roughness on the film surface. In addition, plasma active species may lower the surface hydrophobicity by increasing the number of polar anchors on the surface [42]. Based on a previous study by Wong et al. [43], LDPE films increased in tensile strength parameters due to an increase in plasma treatment time, which was caused by the formation of interfacial roughness and polar groups. The process of coating the active ingredient is easier on the surface of the film with high roughness. This study also compared the surface morphology of the control LDPE film (without plasma treatment) with that of the LDPE film with plasma treatment. Wherein, the control film has a uniform and smooth surface. Meanwhile, the film with plasma treatment had an increase in roughness due to the plasma treatment producing etched characters with irregularly shaped textures. In another study by Theapsak et al. [30] plasma treatment can modify the surface of the PE film by producing oxygen containing polar functional groups (OH, C−O, and C=O) and increasing the surface roughness.

Plasma treatment can increase the binding affinity of MFEO with LDPE film. This is because plasma treatment can cause conformational changes. Based on a previous study, through exposure to hydrophilic groups, plasma treatment can increase the affinity of LDPE film for chitosan, hence boosting the effect of chitosan and gallic acid on LDPE [26,43]. In another study by Loke et al. [27], plasma treatment of LDPE film which was then coated with gallic acid and chitosan showed that the structure of LDPE could not be damaged by plasma, but there was an increase in the affinity of LDPE film for chitosan through exposure to hydrophilic groups. In addition, collagen has a polarity and function closer to cinnamaldehyde, which has better affinity, and more essential oils are retained in the film. In addition, with the use of Pickering emulsion film, there was a decrease in the hydrophobicity of EO, which led to an increase in its affinity (as a hydrophilic material) with the film [44].

### 3.3. Physical and Mechanical Properties

The physical and mechanical properties of the LDPE films prepared in this study are presented in Table 3. The physical properties of the film examined were shear strength (SS), tensile strength (TS), thickness, WVP, and OP. The SS and TS of the film increased as the MFEO concentration increased; a significant difference was found for PE (*p* < 0.05), greater than the increase observed for the CE and NE films. The SS and TS parameters have the same pattern, where the PE 6% treatment has the highest value and is significantly different from the other treatments. Based on previous studies, plasma treatment can reduce TS in the LDPE films [27]. The TS value in the LDPE film was 18.19 MPa, but after plasma treatment, the TS value decreased to 13.71 MPa. In addition, the increase in plasma treatment power of LDPE film had no effect on TS compared with LDPE film without plasma treatment. However, increasing plasma treatment time had a significant effect on decreasing TS in LDPE films. This phenomenon is caused by increased roughness and the formation of polar groups. In addition, prolonged exposure to plasma causes slower aging effects in samples. Increased surface roughness makes preventive coating easier [43]. In Table 3, it can be seen that CE and NE treatment can reduce the TS of LDPE film, although the NE 6% treatment has an increase in TS. This is consistent with previous studies in which the addition of NE to WPI-based films reduces the tensile strength of the films caused by the plasticizing effect of NE droplets so that it can weaken the intermolecular interactions between polymer chains [1]. In another study, the addition of rosemary essential oil to carboxymethyl cellulose-polyvinyl alcohol blend films resulted in a film with a decrease in film strength. This was attributed to the effect of EO plasticization on the film structure [45].

The LDPE film with PE treatment had a higher TS value than the CE and NE treatment, but even at PE 6% treatment, it was not significantly different from the control film. Based on previous studies, the addition of PE from SiO_2_ nanoparticles and functional oil phase resulted in an increase in the tensile strength of the epoxy composite compared with the reference sample [46]. The addition of cellulose nanofiber to sandalwood oil Pickering emulsion can increase the tensile strength of the film. It is associated with the formation of a rigid continuous network of cellulose nanofibers linked through hydrogen bonds, and is also attributed to the geometry and rigidity of the nano-filler [47].

The thickness parameter for the PE treatment shows a significant increase (*p* < 0.05) compared with the CE and NE treatments (Table 3). The thickness parameter for CE and NE films was not significantly different except for NE 6% films. In the study of Fasihi et al. [45], the increase in thickness after the addition of a PE was proposed to be due to the increased solid material content of the film. This was in accordance with the results of our study, the CE and NE treatments did not have a significant effect on thickness.

As shown in Table 3, increasing the concentration of MFEO reduced the WVP of film (*p* < 0.05). Treatment of CE, NE, and PE films for all MFEO concentrations had lower WVP values than control films. According to Ghadetaj et al. [1] the hydrophobic nature of lipid compounds from MFEO can reduce WVP, where the film with CE treatment absorbs less than the film with NE. This is related to the nano size which can limit the droplet size and reduce its plasticizing effect on the film. Different types of emulsion type result in different WVP values; the CE treatment resulted in a significantly lower value than the NE or PE treatments (*p* < 0.05). The study by Fasihi et al. [45] showed that the addition of 5% PE from rosemary essential oil, can reduce WVP in the carboxymethyl cellulose-polyvinyl alcohol blend film. This is attributed to the PE providing decreased water vapor diffusion due to increased tortuous paths. In a previous study, the use of PE in the chitosan matrix interfered with the formation of hydrogen bonds between chitosan molecules and weakened the chitosan network in the film, which facilitated the migration of water vapor molecules [13].

In Table 3, the control group had a significantly (*p* < 0.05) higher OP than the treated film. The addition of MFEO decreased the OP of the LDPE film. OP parameters can be seen NE 6% is the lowest OP value. In another study, cinnamaldehyde increased the oxygen barrier, resulting in a decrease in OP [48], which was possibly a result of the antioxidant properties of cinnamaldehyde, which conferred the oxygen-capturing capacity [49]. The PE film can reduce the OP parameter, it can be seen that PE film has a lower OP than the control film. In a previous study, the loading of zein/chitosan-stabilized PEs increased the oxygen barrier properties of the films. This is because the zein/chitosan-stabilized PEs film has a thick and firm network so that it can act as a natural barrier to oxygen. In addition, the increase in the barrier properties of the chitosan film was related to the delicate interactions between the Pickering emulsion and the chitosan matrix [13].

**Figure 1:**
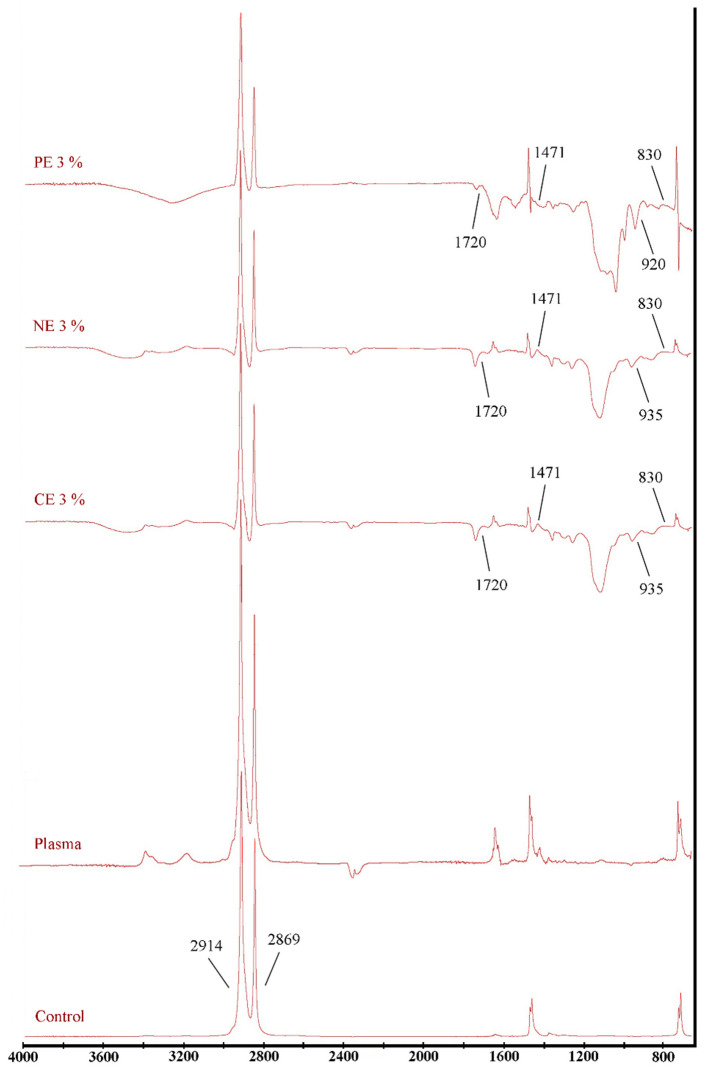
ATR–FTIR of LDPE film treated with cold plasma containing CE-, NE-, and PE-stabilized MFEO.

The infrared spectra of the films shown in Figure 1 indicate the specific functional groups and their vibrational modifications after cold plasma treatment. This analysis was performed only on films treated with 3% MFEO. FTIR showed that there were differences between control films and plasma-treated films including films that were coated with CE, NE, and PE. In the film, with the addition of MFEO, new peaks appear and can be seen in the band between 800 and 1800 cm^−1^. The CE and NE films have quite similar trends because the two emulsions only have size differences, while for PE films there are significant differences (Figure 1). The film with MFEO coating shows the addition of a peak, thus indicating that the active compound from MFEO was successfully added to the LDPE film. The MFEO spectrum contained a high number of peaks, indicating the existence of a variety of volatile compounds and functional groups. In a previous study, Silva-Damasceno et al. [50] found that sharp peaks of MFEO consisted of peaks at 775 and 824 cm^−1^ (aromatic–CH bending), 922 cm^−1^ (–OH bending), 1037 cm^−1^ (–CH2 group vibration), 1238 cm^−1^ (carbonyl vibration), 1507 cm^−1^ (–CH bending), 1715 cm^−1^ (C=C stretching at aromatic groups), 2920 cm^−1^ (–CH stretching), and 3455 cm^−1^ (–OH stretching). The incorporation of MFEO into the matrix led to minor changes in the wavenumber of different peaks, as well as increased peak intensity at 1107 cm^−1^ (P=O), 1418 cm^−1^ (–CH stretching), 1554 cm^−1^ (–NH bending), and 2920–3411 cm^−1^ (broadening of–CH stretching). The flavonoid functional groups correspond to aromatic ring vibrations [51]. Functional groups of flavonoids are located in the range of 1610–1600 cm^−1^ and 1480–1450 cm^−1^ [52]. Based on previous studies, the plasma-treated gallic acid film resulted in hydroxyl stretching of the film where the –OH bond showed a radical scavenging capacity which may have antioxidant properties [26].

In a previous study related to the FT-IR spectrum of polyvinyl alcohol and carboxymethyl cellulose films combined with different concentrations of rosemary essential oil, the IR spectrum pattern was not significantly different, indicating that during the incorporation of rosemary essential oil into the film matrix, the structure of the film matrix did not change [45]. With the addition of PE to the film, there was a shift at the peak of 1037 cm^−1^ compared with CE and NE films. Based on previous studies, increasing the content of chitosan and basil oil in the Poly (L-Lactic Acid) film resulted in a decrease in the characteristic peak of the FT-IR plot, possibly due to the film’s low transmittance in the wave number range of 1050–1300 cm^−1^. Furthermore, no Poly (L-Lactic Acid) peak shift was observed in Poly (L-Lactic Acid)-chitosan or Poly (L-Lactic Acid)-chitosan-basil oil films. This suggests that the chemical groups PLLA and CS have no interaction [53]. Figure 1 shows that the PE film has different peaks from CE and NE films, where new peaks appear on PE films at 1548 and 1028 cm^−1^. This is related to WPI, which is used as the core of PE, where peak 1548 cm^−1^ indicates N–H stretching of amide II and 1028 cm^−1^ shows C–N stretching of amines. This is in accordance with the Almasi et al. [16] study, which showed the emergence of new peaks in films using PE. Thus, the presence of additional peaks as well as an increase in the strength of chosen peaks at different wavenumbers was an indication that MFEO was effectively loaded into the matrix.

**Figure 2:**
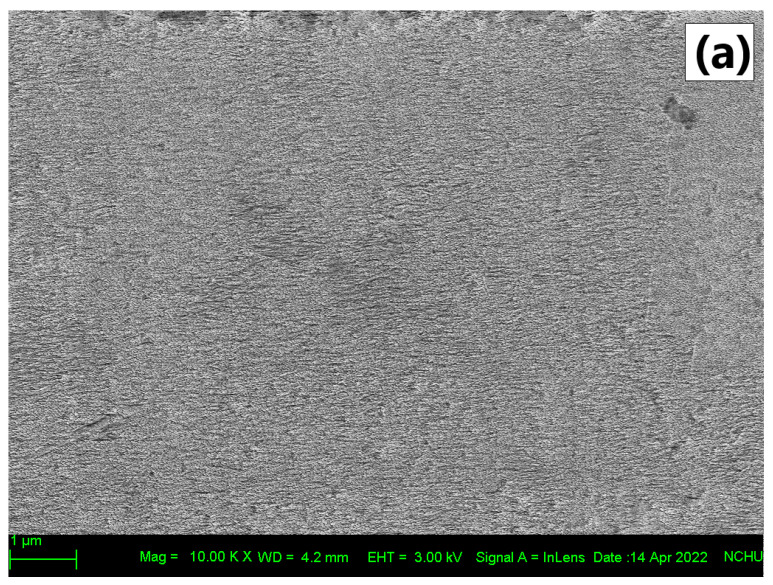

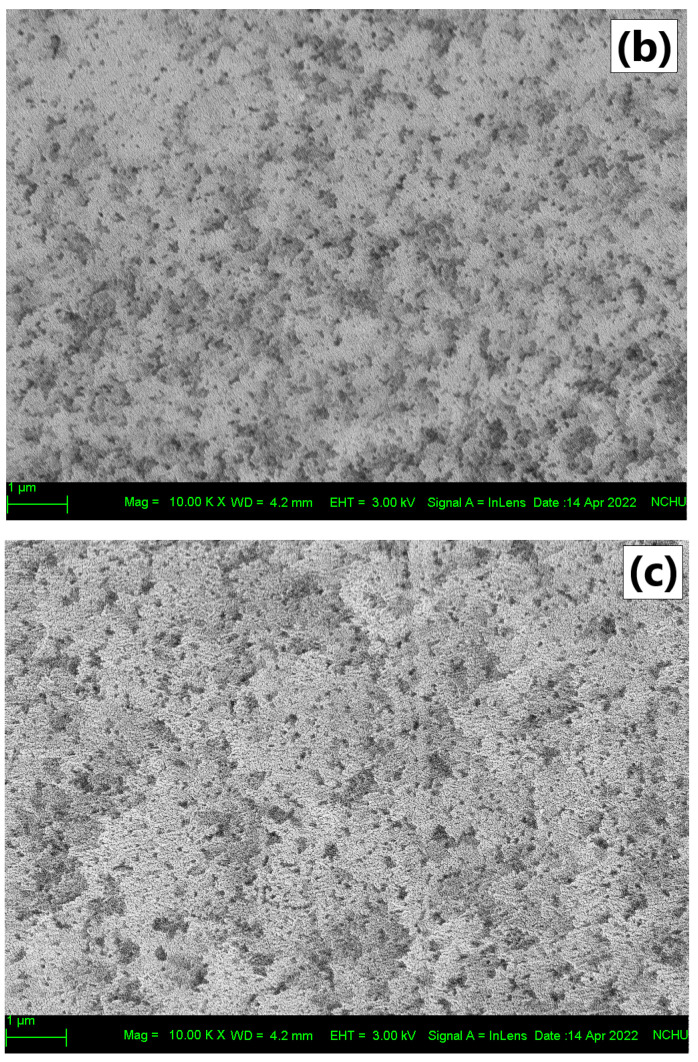

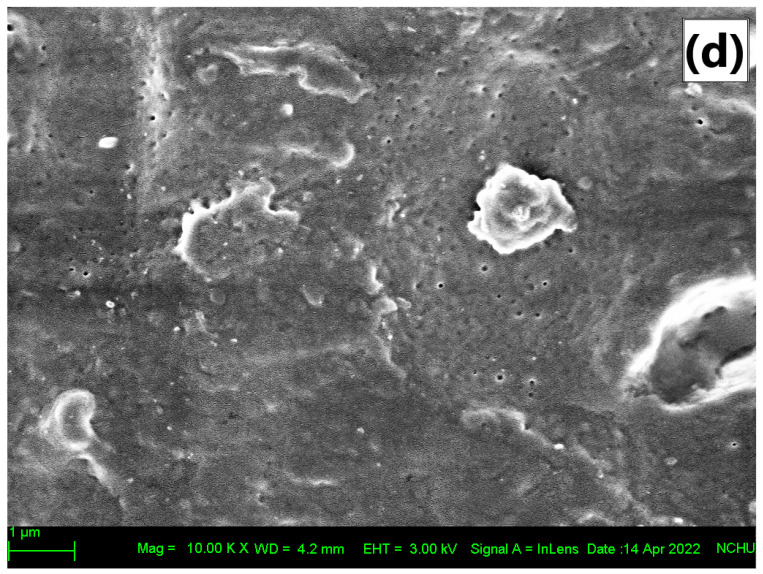
Surface characterization of plasma-treated LDPE film coated with different type of emulsion (**a**) Control (**b**) CE 6% (**c**) NE 6% and (**d**) PE 6% observed under 10,000× magnification.

The characteristic of the LDPE film after cold plasma treatment are presented in Figure 2. This analysis was performed only on films treated with 6% MFEO to determine the differences resulting from the highest concentration of MFEO. The control films had a smooth surface and the LDPE films treated with a CE and NE had rough surface, whereas the film treated with PE appeared ‘sandy’, with fine granules (Figure 2a–d). Surfaces with granules can increase the roughness of the film. In a previous study, the use of pectin films with a concentration of 7.5% PE increased the roughness of the film. This increase in roughness is related to the migration of aggregates or droplets to the top of the film during film drying, thereby causing surface irregularities [16]. In a previous study, the application of cold plasma and cinnamaldehyde-containing CMC coating resulted in the formation of micropores and cavities on the film, which allowed cinnamaldehyde to evaporate during the drying process. The surface of the modified film when combined with polymer compounds can increase EO loading [27]. Plasma treatment can improve interfacial adhesion and polymer matrix compatibility [26].

### 3.4. Antioxidant Properties

**Figure 3:**
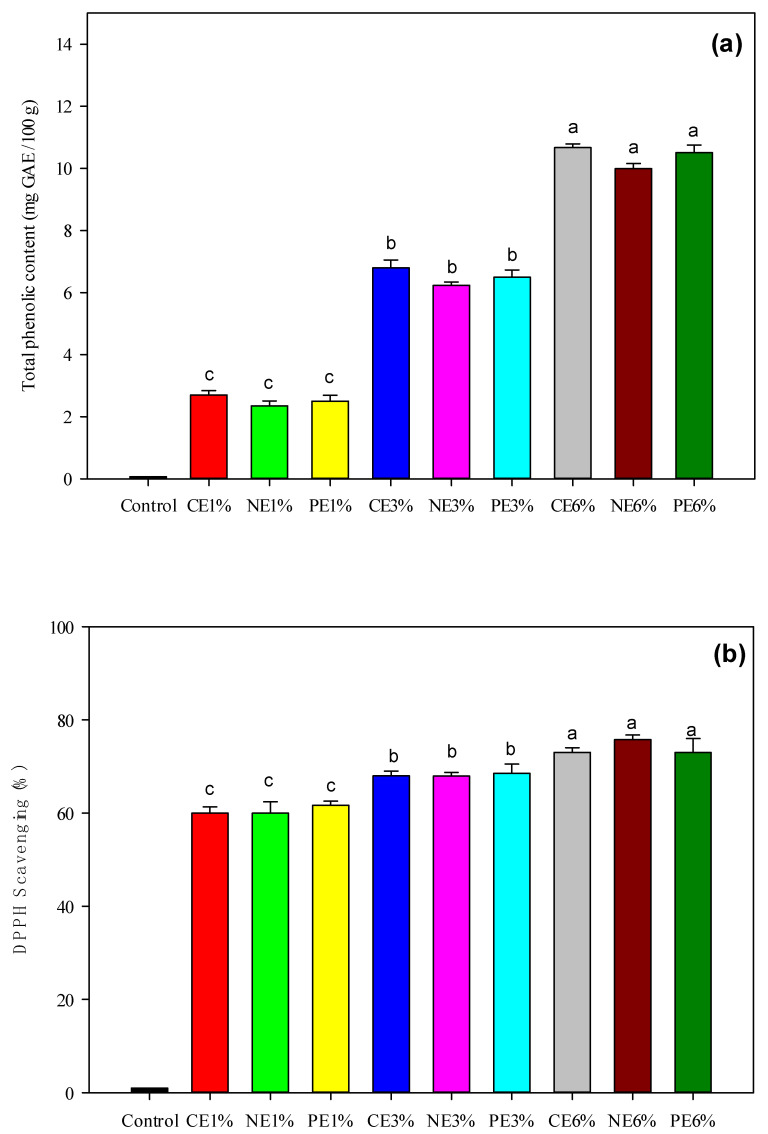
Effectiveness of LDPE film treated with cold plasma containing CE-, NE-, and PE-stabilized MFEO. (**a**) Total phenolic content; (**b**) antioxidant activity through the DPPH radical scavenging assay. ^a–c^ The values with different superscripts are significantly different at *p* < 0.05. The error bars represent the standard deviations (*n* = 3).

As shown in Figure 3, increasing the MFEO concentration significantly (*p* < 0.05) increased the total phenolic content (TPC) and antioxidant activity (DPPH scavenging activity) of the films. TPC and antioxidant activity have a close relationship with EO concentration, and the phenolic group has an important role in antioxidant activity. MFEO contains sabinene, α-pinene, β-pinene, limonene [6], myristicin, and safrole and it contains high phenolic content [7].

Nutmeg essential oil was analyzed using GC-MS and revealed 27 components. myristicin, terpineol-4, alpha-terpinol, dodecanoic acid, torreyol, palmitin, and safrol are some of the compounds found in seeds. Whereas the IC_50_ value of the essential oil of fuli and fruit is higher than that of other nutmeg parts such as seeds, roots, and bark. The IC_50_ values for mace and fruit essential oils are 185,943 ppm and 221,036 ppm, respectively [54]. The antioxidant activity of CE incorporated film samples was not significant different (*p* < 0.05) with NE and PE in the same concentration. In this phenomenon, phenolic acids and terpenoids were found to be responsible for the emulsion, including the film’s DPPH scavenging properties [16]. The antioxidant activity in this study is stronger than in Shokri et al. [37], maximum antioxidant activity was only 30.17% on film containing 3% CE or NE of *Ferulago angulata* EO. The NE films displayed fast and efficient free radical absorption because the formation of NE results in an increase in the specific surface area [25]. In addition, PE films result in the low mobility of the loaded compounds [55] and their slower release is associated with lower antioxidant activity [1]. The antioxidant properties of EO related to the redactors contained in EO can stop and stabilize radical chain reactions. However, it is difficult to trace the antioxidant activity of the whole EO to one or a few active molecules because both minor and major constituents must be taken into account to account for its biological action [56].

### 3.5. Anti-Bacterial Assay

**Figure 4:**
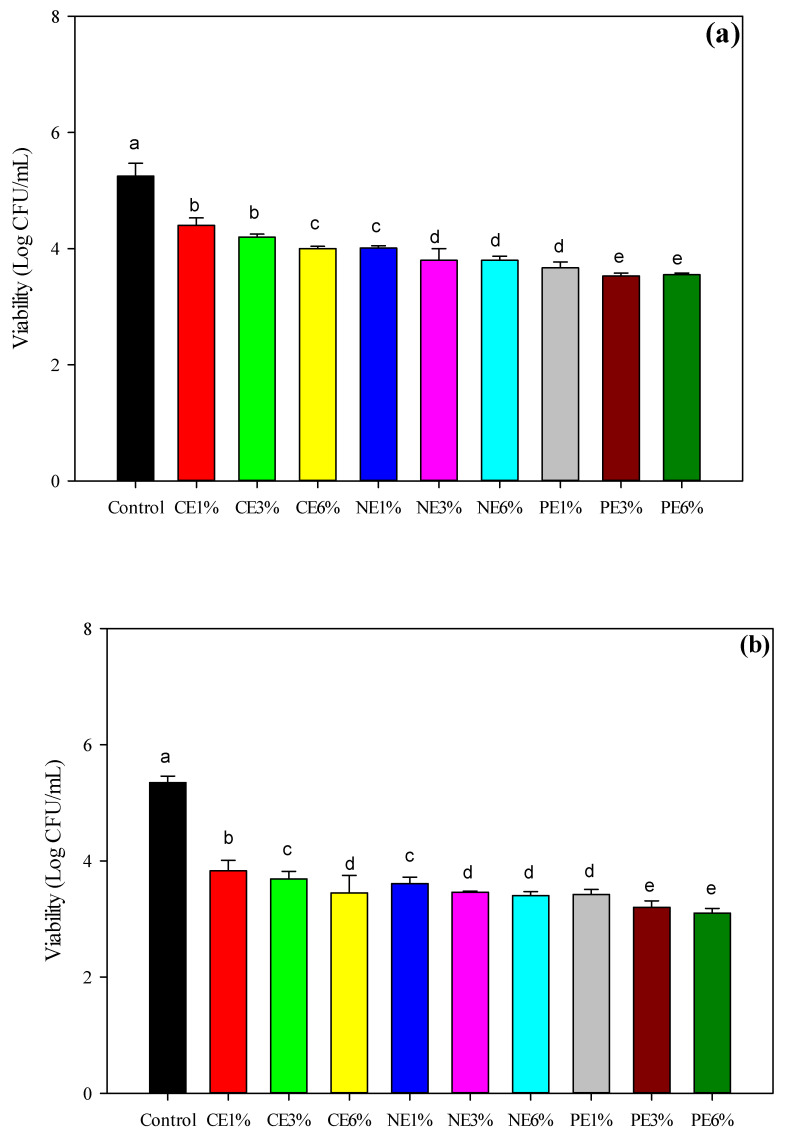
Effectiveness of LDPE film treated with cold plasma containing CE-, NE-, and PE-stabilized MFEO. (**a**) antimicrobial assay against *S. aureus*, (**b**) antimicrobial assay against *E. coli*. ^a–e^ The values with different superscripts are significantly different at *p* < 0.05. The error bars represent the standard deviations (*n* = 3).

As shown in Figure 4, the MFEO film caused a reduction in *E. coli* and *S. aureus* of up to 3.25−4.01 log CFU/mL. In general, there were slight differences in the bacterial inhibition of the CE, NE, and PE treatments, and the reduction in *E. coli* was greater than that in *S. aureus* (*p* < 0.05). An increase in the concentration of MFEO led to an increase in bacterial inhibition, which was indicated by the lower number of bacteria. In addition, MFEO has bacteriostatic properties that can inhibit bacteria and yeasts, including *Arizona*, *Salmonella*, *Enterobacter*, *E. coli*, *Klebsiella pseudomonas*, *S. aureus*, and *Aspergillus flavus*, although the highest inhibitory effectiveness is against *E. coli* [8]. In a previous study, *S. aureus* and *E. coli* were reduced to 4.61–5.14 log CFU/mL after plasma treatment of a cinnamaldehyde coating on LDPE [27].

The inhibition of microbial growth by NE occurs in a variety of ways depending on the encapsulated antimicrobial agents (e.g., EOs, proteins, and surfactants) and the structure of the NE droplets (e.g., composition, charge, and size) [57]. In a previous study, the antibacterial activities of *Origanum majorana* EO were attributed to α-pinene, γ-terpinene, and sabinene [58]. These compounds improved the permeability and fluidity of fungal cells. Terpenes are considered to cause changes in cell permeability by penetrating the fatty acyl chains that comprise the membrane lipid bilayers, altering lipid packing and inducing changes in the membrane functions and properties [1,16]. The increase in anti-bacterial activity depends on the concentration of EO. In addition, the nanoemulsification process in EO can also increase anti-bacterial activity, where the smaller droplet size provides faster penetration of antimicrobial compounds through the bacterial cell membrane [37].

The inhibitory effects of CE and NE treatment were slightly different but were significantly different from the PE film (*p* < 0.05). Nanoemulsification increased the antibacterial activity of coating solutions [37]. The PE films resulted in a significantly slower release than NE, which was the cause of the low antibacterial activity [16]. However, within a short storage time, controlled release, such as NE, may reduce their functional activity [1]. Due to the protective effects of the stable interfacial film, the PE exhibited greater antibacterial activity [45] and could release the active compounds for a longer time.

### 3.6. Release Properties

**Figure 5:**
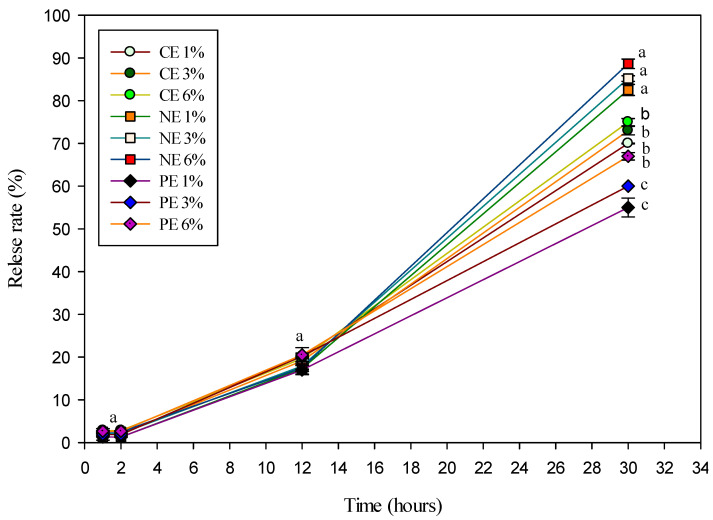
Release rate of LDPE film treated with cold plasma containing CE-, NE-, and PE-stabilized MFEO in 95% alcohol. Values with different superscripts are significantly different at *p* < 0.05. The error bars represent the standard deviation (*n* = 3).

In order to determine the film’s possible application, the release capacity and corresponding release mechanism of active substances loaded in the film matrix were investigated. As shown in Figure 5, there was a slight significant difference in the release properties for all films (*p* < 0.05) after 30 h. In general, the release rate of PE films was lower than that of CE and NE films. After 2 h, similar trends were found for all film samples. This is similar to Ghadetaj et al. [1], who reported that WPI-based films containing NE-loaded EOs had antioxidant activity that was not significantly different from films containing free EOs. The release of compounds loaded in PE films is slower than NE films, resulting in the reduced mobility of loaded compounds [55]. However, over a long storage time, the release of PE is enhanced and causes the retention of their functional activity in a variety of environments [2]. The PE-treated film had lower antioxidant activity than the NE-treated film owing to the slower release of the encapsulated MEO from the film matrix [16]. The PE is stabilized by the WPI-inulin complex, which is absorbed at the oil-water phase interface, thereby shielding MEO from external factors and inhibiting the coalescence of EO [1]. The decrease in the release rate and anti-bacterial activity in the PE film in the short term is possible due to the decrease in the diffusivity of the MFEO in the film matrix [44].

Higuchi and Korsmeyer–Peppas models were utilized to further define the in vitro release properties of the active compounds in loaded films. The correspondence between the actual active compounds released is indicated by the R value close to 1. The previous study by Ritger and Peppas [59], showed that when *n* > 1 the composite Case-II transport mechanism was valid; when 0.5 < *n* < 1 the diffusion behavior followed non-Fickian diffusion; and when *n* < 0.5 the diffusion behavior of the active ingredient in the film matrix follows Fickian diffusion.

The correlation coefficients of all films generated in this investigation were desirable when applying the Higuchi equation (R^2^ = 0.9687–0.9912) (Table 4). The release of active compounds in the Higuchi model is based on Fick’s laws of diffusion, with the assumption that the matrix’s swelling and dissolution are minor or non-existent, and that the matrix exhibits square root time dependency. The Korsmeyer–Peppas equation was also utilized to confirm and explain the aforesaid findings [24]. Based on the values in Table 4, it shows that the opening of all films was based on the Fickian diffusion pattern. In addition, the release of active compounds from the film structure can be encouraged due to the larger concentration difference between the inner and outer environment [24].

## 4. Conclusions

Cold plasma treatment can improve the properties of LDPE films by facilitating MFEO coating, it is supported by the FTIR results that showed the differences between the control film and plasma-treated film and showed the presence of a new group of active compounds from MFEO on the LDPE coating. The use of different types of emulsion causes different characteristics of the film; in general, the use of CE and NE results in better optical characteristics than PE. Increasing the concentration of MFEO provides increased antioxidant activity and inhibition of bacteria. However, PE has more stability and improved controlled release, where PE can inhibit coalescence of MFEO. PE is suitable, especially over long-term storage. There needs to be more research on how to use emulsions in cold plasma treatment protocols and how to apply them to food.

## Figures and Tables

**Table 1:** The droplet size, zeta potential, and polydispersity index of MFEO emulsion prepared with different concentration and emulsion type.

Emulsion	Droplet Size (nm)	Zeta Potential (mV)	Polydispersity Index
CE 1%	1533.15 ± 0.31 ^a^	40.80 ± 0.25 ^a^	0.66 ± 0.37 ^a^
CE 3%	1615.70 ± 0.13 ^a^	38.38 ± 0.29 ^b^	0.81 ± 0.32 ^a^
CE 6%	1731.23 ± 0.24 ^a^	26.54 ± 0.58 ^c^	0.93 ± 0.11 ^a^
NE 1%	170.93 ± 0.11 ^b^	8.47 ± 0.26 ^d^	0.22 ± 0.01 ^c^
NE 3%	197.46 ± 0.52 ^b^	12.10 ± 0.01 ^c,d^	0.38 ± 0.02 ^b^
NE 6%	155.60 ± 0.10 ^b^	11.13 ± 0.03 ^c,d^	0.25 ± 0.02 ^c^
PE 1%	244.76 ± 0.86 ^b^	21.30 ± 0.26 ^c^	0.39 ± 0.01 ^b^
PE 3%	236.98 ± 0.07 ^b^	18.34 ± 0.10 ^c,d^	0.29 ± 0.04 ^c^
PE 6%	233.56 ± 0.11 ^b^	19.43 ± 0.08 ^c,d^	0.32 ± 0.04 ^b,c^

^a–d^ The values in the table are the average ± standard error of *n* = 3 samples and the different lowercase letter in each column indicate significant differences (*p* < 0.05). CE: coarse emulsion; NE: nanoemulsion; PE: Pickering emulsion.

**Table 2:** Optical characteristics of LDPE film-treated cold plasma-stabilized MFEO.

Emulsion	*L*	*a*	*b*	ΔE	*YI*	Opacity	WCA
Control	93.01 ± 0.10 ^a^	0.10 ± 0.16 ^a^	−6.76 ± 0.16 ^d^	-	−10.38 ± 0.16 ^e^	0.45 ± 0.16 ^f^	4.88 ± 0.01 ^e^
CE 1%	27.79 ± 0.16 ^b,c^	−11.80 ± 0.19 ^e^	2.35 ± 0.18 ^c^	1.70 ± 0.16	12.10 ± 0.18 ^d^	1.05 ± 0.04 ^e^	6.22 ± 0.01 ^d^
CE 3%	24.76 ± 0.55 ^c^	−10.09 ± 0.09 ^c^	2.88 ± 0.88 ^c^	0.08 ± 0.88	16.40 ± 0.49 ^c^	1.07 ± 0.05 ^e^	7.63 ± 0.01 ^b^
CE 6%	25.20 ± 0.35 ^c^	−9.47 ± 0.15 ^b^	3.15 ± 0.61 ^c^	0.35 ± 0.31	17.89 ± 0.35 ^b,c^	1.06 ± 0.15 ^e^	7.73 ± 0.01 ^b^
NE 1%	25.20 ± 0.13 ^c^	−10.78 ± 0.18 ^d^	4.22 ± 0.29 ^b^	0.31 ± 0.04	23.92 ± 0.08 ^b^	1.10 ± 0.05 ^d^	4.94 ± 0.05 ^d,e^
NE 3%	31.21 ± 0.65 ^b^	−11.01 ± 0.50 ^d^	6.58 ± 0.40 ^a^	0.49 ± 0.71	30.10 ± 0.66 ^a^	1.15 ± 0.05 ^c^	5.72 ± 0.10 ^d^
NE 6%	30.95 ± 0.21 ^b^	−12.18 ± 0.07 ^e^	6.77 ± 0.10 ^a^	0.36 ± 0.21	31.24 ± 0.21 ^a^	1.15 ± 0.06 ^c^	8.83 ± 0.09 ^a^
PE 1%	27.05 ± 0.55 ^b,c^	−13.70 ± 0.55 ^g^	5.91 ± 0.14 ^a^	0.50 ± 0.22	21.55 ± 0.60 ^b^	1.46 ± 0.47 ^b^	4.66 ± 0.05 ^e^
PE 3%	26.72 ± 0.49 ^b,c^	−12.86 ± 0.33 ^f^	4.19 ± 0.69 ^b^	0.40 ± 0.43	22.57 ± 0.55 ^b^	1.44 ± 0.10 ^b^	6.62 ± 0.01 ^c^
PE 6%	31.24 ± 0.02 ^b^	−11.68 ± 0.12 ^e^	4.71 ± 0.54 ^b^	0.40 ± 0.03	31.42 ± 0.04 ^a^	2.34 ± 0.05 ^a^	8.28 ± 0.07 ^a^

^a–g^ The values in the table are the average ± standard error of *n* = 3 samples and the different lowercase letter in each column indicate significant differences (*p* < 0.05). CE: coarse emulsion; NE: nanoemulsion; PE: Pickering emulsion.

**Table 3:** Physical and mechanical properties of LDPE film-treated cold plasma-stabilized MFEO.

Emulsion	SS (Mpa)	TS (Mpa)	Thickness	WVP	OP
			(mm)	(×10^−7^ g·m^−1^·s^−1^·Pa^−1^)	(×10^−12^ g·m·m^−2^·s^−1^·Pa^−1^)
Control	20.0 ± 0.26 ^a^	18.3 ± 0.25 ^a^	0.034 ± 0.03 ^b^	2.45 ± 0.01 ^a^	6.16 ± 0.01 ^a^
CE 1%	15.5 ± 0.26 ^d^	18.6 ± 0.34 ^a^	0.034 ± 0.01 ^b^	1.47 ± 0.01 ^b^	4.50 ± 0.01 ^b^
CE 3%	15.5 ± 0.28 ^d^	12.3 ± 0.25 ^d^	0.034 ± 0.00 ^b^	1.41 ± 0.06 ^c^	4.37 ± 0.01 ^b^
CE 6%	14.5 ± 0.28 ^e^	11.5 ± 0.28 ^d^	0.034 ± 0.10 ^b^	1.39 ± 0.01 ^c^	4.42 ± 0.01 ^b^
NE 1%	14.3 ± 0.01 ^e^	11.3 ± 0.01 ^d^	0.034 ± 0.01 ^b^	1.46 ± 0.01 ^b^	4.98 ± 0.01 ^b^
NE 3%	14.9 ± 0.26 ^d^	11.8 ± 0.01 ^d^	0.035 ± 0.00 ^b^	1.45 ± 0.01 ^b^	3.18 ± 0.01 ^c^
NE 6%	15.1 ± 0.05 ^d^	18.9 ± 0.05 ^a^	0.038 ± 0.02 ^a^	1.20 ± 0.01 ^c^	2.37 ± 0.01 ^d^
PE 1%	17.0 ± 0.28 ^c^	13.4 ± 0.12 ^c^	0.035 ± 0.01 ^b^	1.46 ± 0.01 ^b^	3.32 ± 0.01 ^c^
PE 3%	19.4 ± 0.23 ^b^	15.3 ± 0.01 ^b^	0.039 ± 0.11 ^a^	1.52 ± 0.09 ^b^	3.04 ± 0.01 ^c^
PE 6%	23.3 ± 0.40 ^a^	18.4 ± 0.02 ^a^	0.039 ± 0.01 ^a^	1.59 ± 0.20 ^b^	3.05 ± 0.01 ^c^

^a–e^ The values in the table are the average ± standard error of *n* = 3 samples and the different lowercase letter in each column indicate significant differences (*p* < 0.05). CE: coarse emulsion; NE: nanoemulsion; PE: Pickering emulsion; SS: Shearing strength; TS: Tensile strength; WVP: Water vapor permeability; OP: Oxygen permeability.

**Table 4:** Parameters of Higuchi/Korsmeyer–Peppas model for release properties of LDPE film treated with cold plasma containing CE-, NE-, and PE-stabilized MFEO.

Emulsion	Higuchi		Korsmeyer–Peppas		
	K_1_	R^2^	K_2_	n	R^2^
CE 1%	2.5627	0.9727	1.1136	0.1334	0.9805
CE 3%	2.5829	0.989	1.0464	0.2295	0.9816
CE 6%	2.6465	0.9843	1.0669	0.1872	0.9721
NE 1%	2.8933	0.9809	1.1831	0.0707	0.9643
NE 3%	2.8512	0.9687	1.2052	0.0236	0.9638
NE 6%	2.8522	0.9844	1.0586	0.2529	0.9659
PE 1%	3.1184	0.9894	1.0212	0.0348	0.9968
PE 3%	2.9334	0.9862	1.1089	0.2674	0.9859
PE 6%	2.49	0.9912	1.0225	0.4515	0.9746

CE: coarse emulsion; NE: nanoemulsion; PE: Pickering emulsion.

## Data Availability

All the data are available within the manuscript.

## References

[1] Ghadetaj A., Almasi H., Mehryar L. (2018). Development and characterization of whey protein isolate active films containing nanoemulsions of *Grammosciadium ptrocarpum* Bioss. essential oil. Food Packag. Shelf Life.

[2] Li J., Xu X., Chen Z., Wang T., Lu Z., Hu W., Wang L. (2018). Zein/gum Arabic nanoparticle-stabilized Pickering emulsion with thymol as an antibacterial delivery system. Carbohydr. Polym..

[3] Gavahian M., Farahnaky A., Javidnia K., Majzoobi M. (2012). Comparison of ohmic-assisted hydrodistillation with traditional hydrodistillation for the extraction of essential oils from *Thymus vulgaris* L. Innov. Food Sci Emerg. Technol..

[4] Atarés L., Chiralt A. (2016). Essential oils as additives in biodegradable films and coatings for active food packaging. Trends Food Sci. Technol..

[5] D’Souza S.P., Chavannavar S.V., Kanchanashri B., Niveditha S.B. (2017). Pharmaceutical Perspectives of Spices and Condiments as Alternative Antimicrobial Remedy. J. Evid.-Based Complement. Altern. Med..

[6] Matulyte I., Marksa M., Ivanauskas L., Kalvenien Z., Lazauskas R., Bernatoniene J. (2019). GC-MS analysis of the composition of the extracts and essential Oil from *Myristica fragrans* Seeds Using Magnesium Aluminometasilicate as Excipient. Molecules.

[7] El-Alfy A.T., Abourashed E.A., Patel C., Mazhari N., An H.R., Jeon A. (2019). Phenolic compounds from nutmeg (*Myristica fragrans* Houtt.) inhibit the endocannabinoid-modulating enzyme fatty acid amide hydrolase. J. Pharm. Pharmacol..

[8] Mousavi S.M., Hashemi S.A., Ramakrishna S., Esmaeili H., Bahrani S., Koosha M., Babapoor A. (2019). Green synthesis of supermagnetic Fe_3_O_4_–MgO nanoparticles via Nutmeg essential oil toward superior anti-bacterial and anti-fungal performance. J. Drug Deliv. Sci. Technol..

[9] Balakrishnan S., Sivaji I., Kandasamy S., Duraisamy S., Kumar N.S., Gurusubramanian G. (2017). Biosynthesis of silver nanoparticles using *Myristica fragrans* seed (nutmeg) extract and its antibacterial activity against multidrug-resistant (MDR) *Salmonella enterica* serovar Typhi isolates. Environ. Sci. Pollut. Res..

[10] Galeano L.Y., Torres V.O., García S.Á. (2018). Evaluation of nutmeg (*Myristica fragrans* Houtt) as active component during storage of bovine loins. Rev. Cienc. Agrícolas.

[11] Liu Q.R., Wang W., Qi J., Huang Q., Xiao J. (2019). Oregano essential oil loaded soybean polysaccharide films: Effect of Pickering type immobilization on physical and antimicrobial properties. Food Hydrocoll..

[12] Burgos N., Mellinas A.C., García-serna E. (2017). Nanoencapsulation of Flavor and Aromas in Food Packaging. Food Packaging.

[13] Shi W.J., Tang C.H., Yin S.W., Yin Y., Yang X.Q., Wu L.Y., Zhao Z.G. (2016). Development and characterization of novel chitosan emulsion films via pickering emulsions incorporation approach. Food Hydrocoll..

[14] Cossu A., Wang M.S., Chaudhari A., Nitin N. (2015). Antifungal activity against *Candida albicans* of starch Pickering emulsion with thymol or amphotericin B in suspension and calcium alginate films. Int. J. Pharm..

[15] Hosseinnia M., Khaledabad M.A., Almasi H. (2017). Optimization of *Ziziphora clinopodiodes* essential oil microencapsulation by whey protein isolate and pectin: A comparative study. Int. J. Biol. Macromol..

[16] Almasi H., Azizi S., Amjadi S. (2020). Development and characterization of pectin films activated by nanoemulsion and Pickering emulsion stabilized marjoram (*Origanum majorana* L.) essential oil. Food Hydrocoll..

[17] Gavahian M., Meng-Jen T., Khaneghah A.M. (2020). Emerging techniques in food science: The resistance of chlorpyrifos pesticide pollution against arc and dielectric barrier discharge plasma. Qual. Assur. Saf. Crops Foods.

[18] Pankaj S.K., Bueno-Ferrer C., Misra N.N., Milosavljević V., O’Donnell C.P., Bourke P., Keener K.M., Cullen P.J. (2014). Applications of cold plasma technology in food packaging. Trends Food Sci. Technol..

[19] Mendes J.F., Norcino L.B., Martins H.H.A., Manrich A., Otoni C.G., Carvalho E.E.N., Piccoli R.H., Oliveira J.E., Pinheiro A.C.M., Mattoso L.H.C. (2020). Correlating emulsion characteristics with the properties of active starch films loaded with lemongrass essential oil. Food Hydrocoll..

[20] González A., Gastelú G., Barrera G.N., Ribotta P.D., Álvarez Igarzabal C.I. (2019). Preparation and characterization of soy protein films reinforced with cellulose nanofibers obtained from soybean by-products. Food Hydrocoll..

[21] Hu Y., Shi L., Ren Z., Hao G., Chen J., Weng W. (2021). Characterization of emulsion films prepared from soy protein isolate at different preheating temperatures. J. Food Eng..

[22] Wang Q., Liu W., Tian B., Li D., Liu C., Jiang B., Feng Z. (2020). Preparation and characterization of coating based on protein nanofibers and polyphenol and application for salted duck egg yolks. Foods.

[23] Jiang B., Wang L., Zhu M., Wu S., Wang X., Li D., Liu C., Feng Z., Tian C. (2021). Separation, structural characteristics and biological activity of lactic acid bacteria exopolysaccharides separated by aqueous two-phase system. LWT.

[24] Tian B., Cheng J., Zhang T., Liu Y., Chen D. (2022). Multifunctional chitosan-based film loaded with hops β-acids: Preparation, characterization, controlled release and antibacterial mechanism. Food Hydrocoll..

[25] Noori S., Zeynali F., Almasi H. (2018). Antimicrobial and antioxidant efficiency of nanoemulsion-based edible coating containing ginger (*Zingiber officinale*) essential oil and its effect on safety and quality attributes of chicken breast fillets. Food Control.

[26] Wong L.W., Loke X.J., Chang C.K., Ko W.C., Hou C.Y., Hsieh C.W. (2020). Use of the plasma-treated and chitosan/gallic acid-coated polyethylene film for the preservation of tilapia (*Orechromis niloticus*) fillets. Food Chem..

[27] Loke X.J., Chang C.K., Hou C.Y., Cheng K.C., Hsieh C.W. (2021). Plasma-treated polyethylene coated with polysaccharide and protein containing cinnamaldehyde for active packaging films and applications on tilapia (*Orechromis niloticus*) fillet preservation. Food Control.

[28] Han H.S., Song K.B. (2021). Noni (*Morinda citrifolia*) fruit polysaccharide films containing blueberry (*Vaccinium corymbosum*) leaf extract as an antioxidant packaging material. Food Hydrocoll..

[29] Grzegorzewski F., Rohn S., Kroh L.W., Geyer M., Schlüter O. (2010). Surface morphology and chemical composition of lamb’s lettuce (*Valerianella locusta*) after exposure to a low-pressure oxygen plasma. Food Chem..

[30] Theapsak S., Watthanaphanit A., Rujiravanit R. (2012). Preparation of chitosan-coated polyethylene packaging films by DBD plasma treatment. ACS Appl. Mater. Interfaces.

[31] Dammak I., de Carvalho R.A., Trindade C.S.F., Lourenço R.V., do Amaral S.P.J. (2017). Properties of active gelatin films incorporated with rutin-loaded nanoemulsions. Int. J. Biol. Macromol..

[32] Zhang L., Liu Z., Wang X., Dong S., Sun Y., Zhao Z. (2019). The properties of chitosan/zein blend film and effect of film on quality of mushroom (*Agaricus bisporus*). Postharvest Biol. Technol..

[33] Blois M.S. (1985). Antioxidant Determinations by the Use of a Stable Free Radical. Nature.

[34] Piñeros-Hernandez D., Medina-Jaramillo C., López-Córdoba A., Goyanes S. (2017). Edible cassava starch films carrying rosemary antioxidant extracts for potential use as active food packaging. Food Hydrocoll..

[35] Nurjanah N., Jacoeb A.M., Asmara D.A., Hidayat T. (2019). Phenol Component of Fresh and Boiled Sea Grapes (*Caulerpa* sp.) From Tual, Maluku. Food Sci. J..

[36] Qin Y., Yang J., Xue J. (2015). Characterization of antimicrobial poly (lactic acid)/poly(trimethylene carbonate) films with cinnamaldehyde. J. Mater. Sci..

[37] Shokri S., Parastouei K., Taghdir M., Abbaszadeh S. (2020). Application an edible active coating based on chitosan- *Ferulago angulata* essential oil nanoemulsion to shelf life extension of Rainbow trout fillets stored at 4 °C. Int. J. Biol. Macromol..

[38] Söğüt E. (2019). Properties of Solvent Cast Polycaprolactone Films Containing Pomegranate Seed Oil Stabilized with Nanocellulose. Turk. J. Agric.-Food Sci. Technol..

[39] Du M., Sun Z., Liu Z., Yang Y., Liu Z., Wang Y., Jiang B., Feng Z., Liu C. (2022). High efficiency desalination of wasted salted duck egg white and processing into food-grade pickering emulsion stabilizer. LWT.

[40] Yang Y., Jiao Q., Wang L., Zhang Y., Jiang B., Li D., Feng Z., Liu C. (2022). Preparation and evaluation of a novel high internal phase Pickering emulsion based on whey protein isolate nanofibrils derived by hydrothermal method. Food Hydrocoll..

[41] Galus S. (2018). Functional properties of soy protein isolate edible films as affected by rapeseed oil concentration. Food Hydrocoll..

[42] Dong S., Gao A., Zhao Y., Li Y.T., Chen Y. (2017). Characterization of physicochemical and structural properties of atmospheric cold plasma (ACP) modified zein. Food Bioprod. Process..

[43] Wong L.W., Hou C.Y., Hsieh C.C., Chang C.K., Wu Y.S., Hsieh C.W. (2020). Preparation of antimicrobial active packaging film by capacitively coupled plasma treatment. LWT.

[44] Almasi H., Zandi M., Beigzadeh S., Haghju S., Mehrnow N. (2016). Chitosan films incorporated with nettle (*Urtica dioica* L.) extract-loaded nanoliposomes: II. Antioxidant activity and release properties. J. Microencapsul..

[45] Fasihi H., Fazilati M., Hashemi M., Noshirvani N. (2017). Novel carboxymethyl cellulose-polyvinyl alcohol blend films stabilized by Pickering emulsion incorporation method. Carbohydr. Polym..

[46] Li S. (2020). Effect of Pickering emulsion on the mechanical performances and fracture toughness of epoxy composites. Polym. Adv. Technol..

[47] Wardana A.A., Koga A., Tanaka F., Tanaka F. (2021). Antifungal features and properties of chitosan/sandalwood oil Pickering emulsion coating stabilized by appropriate cellulose nanofiber dosage for fresh fruit application. Sci. Rep..

[48] Muller J., González-Martínez C., Chiralt A. (2017). Poly(lactic) acid (PLA) and starch bilayer films, containing cinnamaldehyde, obtained by compression moulding. Eur. Polym. J..

[49] Bonilla J., Talón E., Atarés L., Vargas M., Chiralt A. (2013). Effect of the incorporation of antioxidants on physicochemical and antioxidant properties of wheat starch-chitosan films. J. Food Eng..

[50] Silva Damasceno E.T., Almeida R.R., de Carvalho S.Y.B., de Carvalho G.S.G., Mano V., Pereira A.C., Guimaraes L.G. (2018). *Lippia origanoides* Kunth. essential oil loaded in nanogel based on the chitosan and ρ-coumaric acid: Encapsulation efficiency and antioxidant activity. Ind. Crops Prod..

[51] Yallapu M.M., Jaggi M., Chauhan S.C. (2010). β-Cyclodextrin-curcumin self-assembly enhances curcumin delivery in prostate cancer cells. Colloids Surf. B Biointerfaces.

[52] Członka S., Strąkowska A., Kairytė A., Kremensas A. (2020). Nutmeg filler as a natural compound for the production of polyurethane composite foams with antibacterial and anti-aging properties. Polym. Test..

[53] Salmas C.E., Giannakas A.E., Baikousi M., Leontiou A., Siasou Z., Karakassides M.A. (2021). Development of Poly (L-Lactic Acid)/Chitosan/Basil Oil Active Packaging Films via a Melt-Extrusion Process Using Novel Chitosan/Basil Oil Blends. Processes.

[54] Ginting B., Maira R.M., Helwati H., Desiyana L.S., Mujahid R. (2018). Isolation of essensial oil of nutmeg (*Myristica fragrans* Houtt) and antioxidant activity test with DPPH. J. Nat..

[55] Dammak I., Lourenço R.V., Sobral P.J.A. (2019). Active gelatin films incorporated with Pickering emulsions encapsulating hesperidin: Preparation and physicochemical characterization. J. Food Eng..

[56] Wang W., Wu N., Zu Y.G., Fu Y.J. (2008). Antioxidative activity of Rosmarinus officinalis L. essential oil compared to its main components. Food Chem..

[57] Li J., Mcclements D.J., Mclandsborough L.A. (2001). Interaction between emulsion droplets and *Escherichia coli* cells. J. Food Sci..

[58] Burt S. (2004). Essential oils: Their antibacterial properties and potential applications in foods—A review. Int. J. Food Microbiol..

[59] Ritger P.L., Peppas N.A. (1987). A simple equation for description of solute release II. Fickian and anomalous release from swellable devices. J. Control. Release.

